# In Vitro Biotransformation, Safety, and Chemopreventive Action of Novel 8-Methoxy-Purine-2,6-Dione Derivatives

**DOI:** 10.1007/s12010-017-2527-z

**Published:** 2017-06-17

**Authors:** Małgorzata Anna Marć, Enrique Domínguez-Álvarez, Karolina Słoczyńska, Paweł Żmudzki, Grażyna Chłoń-Rzepa, Elżbieta Pękala

**Affiliations:** 1 Department of Pharmaceutical Biochemistry, Faculty of Pharmacy, Jagiellonian University Medical College, 9 Medyczna Street, 30-688 Kraków, Poland; 2 Institute of General Organic Chemistry, Spanish National Research Council (IQOG-CSIC), Juan de la Cierva 3, 28006 Madrid, Spain; 3 Department of Medicinal Chemistry, Faculty of Pharmacy, Jagiellonian University Medical College, 9 Medyczna Street, 30-688 Kraków, Poland

**Keywords:** Alternative test, Ames test, *Cunninghamella* assay, DPPH assay, Microsomal stability

## Abstract

Metabolic stability, mutagenicity, antimutagenicity, and the ability to scavenge free radicals of four novel 8-methoxy-purine-2,6-dione derivatives (compounds 1–4) demonstrating analgesic and anti-inflammatory properties were determined. Metabolic stability was evaluated in *Cunninghamella* and microsomal models, mutagenic and antimutagenic properties were assessed using the Ames and the *Vibrio harveyi* tests, and free radical scavenging activity was evaluated with 2,2-diphenyl-1-picrylhydrazyl radical scavenging assay. In the *Cunninghamella* model, compound 2 did not undergo any biotransformation; whereas 3 and 4 showed less metabolic stability: 1–9 and 53–88% of the parental compound, respectively, underwent biotransformation reactions in different *Cunninghamella* strains. The metabolites detected after the biotransformation of 3 and 4 were aromatic hydroxylation and *N*-dealkylation products. On the other hand, the *N*-dealkylation product was the only metabolite formed in microsome assay. Additionally, these derivatives do not possess mutagenic potential in microbiological models (*Vibrio harveyi* and *Salmonella typhimurium*) considered. Moreover, all compounds showed a strong chemopreventive activity in the modified *Vibrio harveyi* strains BB7X and BB7M. However, radical scavenging activity was not the mechanism which explained the observed chemopreventive activity.

## Introduction

During the biotransformation processes, drugs and chemicals are structurally modified by various enzymatic systems to form more polar substances, which can be excreted more easily than the original compounds. Problems arise when these modifications generate toxic products [1, 2]. Traditionally, drug metabolism studies use in vivo experiments in mice, rat or guinea pig, or chimeric mouse models with transplanted human hepatocytes [3, 4]. However, these models can create ethical dilemmas; and the experiments are expensive and time consuming [3]. In addition, metabolites are sometimes produced in low amounts, thus hindering their identification [4].

Enzymatic systems involved in the metabolism of exogenous organic compounds are similar in mammals and in certain fungal microorganisms, like *Cunninghamella*. Therefore, *Cunninghamella* fungi can be used as an alternative to in vivo metabolism models [1–3, 5–8]. The use of these microorganisms enables the reduction of research costs and does not arise ethical dilemmas. These alternative tests are simple and reliable as metabolites can be easily extracted to be identified later using instrumental analytical techniques [3]. *Cunninghamella echinulata* contains a rich set of microsomal cytochrome P450, and it can conduct phase I (oxidative) and phase II (conjugative) biotransformation under certain conditions. Moreover, the fungus possesses the ability to metabolize a wide variety of xenobiotics in regio- and stereo-selective manners [9].

A second alternative to in vivo metabolism assays of chemical compounds are in vitro screening assays with liver microsomes: they can predict the metabolic routes of tested compounds, as well as kinetics of these transformations [10]. Cytochrome P450 enzymes are involved in phase I reactions, whereas phase II reactions are commonly carried out by different transferase enzymes such as glutathione transferase and *N*-acetyltransferase [11]. As microsomal assays have a good reproducibility and performing them is straightforward, they are becoming a standard in vitro screening in drug discovery processes [12–14]. Finally, liver microsomes from different species (such as rat, mouse, dog, monkey, and human) can be used to provide more accurate data [11].

Zygmunt et al. [15, 16] reported in previous studies that some novel derivatives of 8-methoxy-purine-2,6-diones substituted at 7-position of purine core by carboxylic, ester, or amide moieties showed potent anti-inflammatory and analgesic activity, superior to the reference drug, in preliminary pharmacological studies [15, 16]. Their analgesic properties were evaluated in two pharmacological in vivo models: formalin and writhing syndrome tests. On the other hand, anti-inflammatory potential of the aforesaid compounds was determined using zymosan-induced peritonitis and carrageenan-induced edema models. Derivatives containing an amide substituent showed very strong analgesic and anti-inflammatory (antiedematous) activities. The most active compound (4) showed a 23-fold and a 36-fold higher activity than acetylsalicylic acid (the analgesic used in clinical practice considered as reference) in the writhing syndrome test in mice and in the formalin test in mice, respectively [15, 16]. Thus, these newly synthesized derivatives represent a new class of analgesic and anti-inflammatory agents with possible future therapeutic applications in the treatment of inflammatory diseases and in the attenuation of pain. However, once verified their promising activity, more in-depth studies need to be performed in their pharmacology, pharmacokinetics, safety, and toxicology, among other fields, to evaluate the feasibility of the possible clinical applications of these compounds, as well as to know better how they exert their action.

**Fig. 1:**
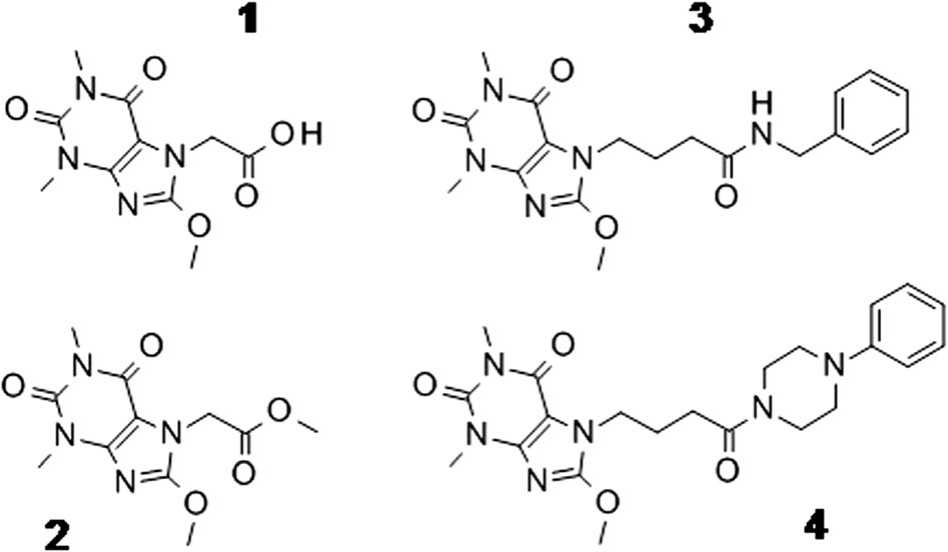
Chemical structures of compounds 1–4

In line to this, the aim of this study was to investigate in vitro biotransformation and some biological activities of the four (1–4) most promising 8-methoxy-purine-2,6-dione derivatives (Fig. 1) selected on the basis of their activity [15, 16]. The activities evaluated in the present study are mutagenicity, antimutagenicity, metabolic biotransformations, and the ability of the compounds to scavenge free radicals. Among the four compounds tested, there are 2-(8-methoxy-1,3-dimethyl-2,6-dioxo-purin-7-yl)acetic acid (1), methyl 2-(8-methoxy-1,3-dimethyl-2,6-dioxo-purin-7-yl)acetate (2), *N*-benzyl-4-(8-methoxy-1,3-dimethyl-2,6-dioxo-purin-7-yl)butanamide (3), and 8-methoxy-1,3-dimethyl-7-[4-oxo-4-(4-phenylpiperazin-1-yl)butyl]purine-2,6-dione (4).

## Materials and Methods

### Tested Compounds

Four 8-methoxy-purine-2,6-dione derivatives (1–4) described in previous publications [15, 16] were provided by Dr. Grażyna Chłoń-Rzepa from the Department of Medicinal Chemistry, Jagiellonian University Medical College. The structures of these compounds were established on the basis of CHN elemental analysis and spectral data (IR, ^1^HNMR, and mass spectra). Additionally, qualitative analysis was performed using TLC and HPLC.

In the *Cunninghamella* biotransformation assay, 12.5 mg of compounds 2–4 were dissolved in 0.5 mL of DMSO to be later inoculated in 24.5 mL of broth medium containing fungus, being thus 0.5 mg/mL the final concentration. For rat microsomal assay, 5.1 mM stock solutions of compounds 1 and 3 were prepared in water and ethanol, respectively. From them, 0.2 mM working solutions were prepared by dilution with water. In mutagenic tests, compounds 1–4 were dissolved in pure DMSO to obtain the corresponding stock solution (10 mg/mL). Working solutions were prepared by 1:100 dilution of stock solution in water, and final compound concentration assayed was 40 ng/mL. Finally, for 2,2-diphenyl-1-picrylhydrazyl (DPPH) assay, 1 mM stock solutions of compounds 1–4 were prepared in methanol.

### Reagents

4-Nitroquinoline *N*-oxide (NQNO), DMSO, l-histidine monochloride, yeast extract, levallorphan, NADP^+^, glucose-6-phosphate sodium salt, glucose-6-phosphate dehydrogenase, potassium hydroxide, DPPH, gallic acid, and ascorbic acid were purchased from Sigma-Aldrich (Seelze, Germany); Nutrient Broth No. 2, from Argenta (Poznań, Poland); glycerol and neomycin sulfate, from Pharma Cosmetic (Kraków, Poland); sodium sulfate, sodium chloride, d-glucose, and dipotassium hydrogen phosphate from Chempur (Piekary Śląskie, Poland); dichloromethane from Stanlab (Lublin, Poland); and agar-agar, bacto-peptone, beef extract, potato dextrose agar, and 0.2-mm silica-coated aluminum TLC plates from Merck (Darmstadt, Germany).

### *Cunninghamella* Strains and Culture Conditions

Three different strains of *Cunninghamella* were used: *Cunninghamella echinulata* NRRL 1384, *Cunninghamella blakesleeana* 1908 DMS, and *Cunninghamella elegans* 1906 DMS. The first was provided by Dr. A.J. Carnell (University of Liverpool, UK), whereas the two remaining were purchased from Deutsche Sammlung von Mikroorganismen und Zellkulturen GmbH (DMSZ, Braunschweig, Germany).

*Cunninghamella* strains were cultivated on solid potato dextrose agar (PDA) plates at 28 °C. After 4 days, a liquid fermentation basal medium was prepared by the addition of 6 g of d-glucose, 1.5 g of NaCl, 1.5 g of yeast extract from *Saccharomyces cerevisiae*, and 1.5 g of K_2_HPO_4_ to 300 mL of distilled water. Aliquots of 24.5 mL of medium were inoculated with 300 μL of suspension of fungal spores obtained after wetting colonies in PDA. The fungi were incubated with shaking until the formation of spherical clumps.

### *Cunninghamella* Biotransformation Assay

Compounds 2–4 were evaluated in this experiment. Once microorganisms formed spherical clumps in the preliminary incubation, the corresponding tested compound was added to the broth and the mixture was incubated for 7 days. Two control experiments were also performed, in the absence of compound and fungus, respectively [3, 9]. Biotransformation progress was monitored by TLC and by liquid chromatography electrospray ionization-tandem mass spectrometry (LC-MS/MS). Monitoring was performed at four different incubation times: 30 min, 3 days, 5 days, and 7 days.

To analyze metabolites in LC-MS/MS, an aliquot of 500 μL of filtered sample was taken and extracted with 500 μL of dichloromethane. The organic fraction was dried with sodium sulfate, filtered, and evaporated. The obtained residue was analyzed in LC-MS/MS. At the end of experiment, all the remaining medium was extracted with 25 mL of dichloromethane after separating the fungal clumps by filtration over cellulose filters [3, 17].

### Microsomal Stability Assay

Compounds 1 and 3 were studied in a biotransformation assay using rat liver microsomes following procedures described previously [18–22]. The reagents used in this assay were levallorphan (internal standard) and NADPH-regenerating system, which contained phosphate buffer (pH 7.4), NADP^+^, glucose-6-phosphate, and glucose-6-phosphate dehydrogenase [18]. The final protein concentration in the assay was 0.4 mg/mL. Compounds 1 and 3 were tested at a final concentration of 20 μM.

The amount of a parental compound remaining in a solution after biotransformation was monitored using LC-MS/MS as described previously in “*Cunninghamella* Biotransformation Assay.” Graphs representing ln of percentage of parent compound remaining versus incubation time were drawn to calculate in vitro half-time (*t*_1/2_) from the slope of linear regression. To obtain intrinsic clearance (Cl_int_) of a tested compound in rat liver microsomal assay, *t*_1/2_ previously calculated was substituted at the equation Cl_int_ = [(*V*_mic_/*P*_mic_) × 0.693/*t*_1/2_], being *V*_mic_ and *P*_mic_ the volume of incubation in microliters and the amount of incubated protein in milligrams, respectively [23, 24].

### Analytical Methods

TLC experiments were performed using 0.2-mm silica-coated aluminum plates, being a 95:5 dichloromethane/methanol mixture the mobile phase. The plates were observed under UV light.

Agilent 1100 HPLC system (Agilent Technologies, Waldbronn, Germany) equipped with a Xbridge™ C18 analytical column (2.1 × 30 mm, 3.5 μM; Waters, Dublin, Ireland) was used in the chromatographic separation of LC-MS/MS analysis. An elution gradient of acetonitrile and water, with 0.1% of formic acid, was used as a mobile phase. MS spectra were taken at Applied Biosystems MDS Sciex API 200 triple quadrupole mass analyzer (Concord, Ontario, Canada) with an electrospray ionization (ESI) interface between HPLC and MS.

### Bacterial Strains

Four *Vibrio harveyi* strains were used in this study: a wild-type BB7 (naturally found in the Baltic Sea) and three genetically modified strains, namely BB7M (obtained by the introduction of pAB1273 plasmid containing the genes *mucA* and *mucB* to enhance the error-prone DNA repair), BB7X (hypersensitive to UV radiation after genetic modification with Tn5TpMCS), and BB7XM (containing the two abovementioned modifications) [25–28]. These strains were kindly provided by Prof. G. Węgrzyn (Department of Molecular Biology, University of Gdańsk, Poland).

In the Ames test, *Salmonella typhimurium* TA100 strain was used. This strain has a base-pair substitution that can be reverted by mutations at GC pairs. In addition, it contains mutations at *uvrB-bio* and *rfa* genes to eliminate excision repair mechanisms and to make the bacteria more permeable to chemicals, respectively. Finally, TA100 also includes pKM101 plasmid, which enhances both chemical and UV-induced mutagenesis via an increase in the error-prone recombinational DNA repair pathway [29, 30]. TA100 strain of *S. typhimurium* was provided by Dr. T. Nohmi (Division of Genetics and Mutagenesis, National Institute of Hygienic Sciences, Tokyo, Japan). *V. harveyi* and *S. typhimurium* bacterial cultures were maintained at 30 and 37 °C, respectively.

### Mutagenic Agent

A standard mutagen NQNO was used as a positive control in mutagenicity assays and as a mutagen added to all probes (except negative controls) in antimutagenicity tests. It causes point mutations at the genome as it induces G:C → A:T transitions in *Vibrio harveyi* strains [27, 31] and in other bacteria such as *Escherichia coli* and *S. typhimurium* [32]. NQNO was initially dissolved in DMSO. Next, its working solution was prepared in sterile water, being 40 ng/mL its final concentration.

### *Vibrio harveyi* and Ames Mutagenicity Assays

To perform the *V. harveyi* mutagenicity test, 10 μL of a working solution of the tested compound (100 μg/mL) were added to *V. harveyi* bacterial culture (OD_600_ = 0.1) in NaCl-containing BOSS liquid medium, being 40 ng/mL the final concentration of the tested compound. The samples were left incubating till OD_600_ increased to 0.3–0.4. Then, an inoculum containing 5 × 10^6^ cells was added to solid BOSS agar plate supplemented with neomycin sulfate at a final concentration of 100 μg/mL. Samples were left incubating 48 h at 30 °C, and revertant colonies were counted manually. A positive control with NQNO and two negative controls (DMSO and blank water probe) were also tested. Each experiment was performed in triplicate, and results were given in terms of mutagenic index (MI), which is the quotient between the number of revertant colonies induced in a test sample and the number of revertants in a negative control [33–35]. A compound is considered mutagenic if MI is above 2.0 or if the number of revertants is higher in compound samples that in NQNO ones [26, 31].

Additionally, two different final concentrations of compounds (40 and 500 ng/mL) were evaluated in the Ames test to confirm the results obtained in the *Vibrio harveyi* assay using a second independent method. Positive (NQNO, concentration of 40 ng/mL) and negative (DMSO) controls were also tested. *S. typhimurium* was cultivated in a fresh medium 12 h prior to a test. To perform mutagenicity assay, 100 μL of an overnight culture (containing approx. 1 × 10^8^ to 2 × 10^8^ bacteria) were inoculated along with 50 μL of a tested compound in 2 mL of top agar supplemented with traces of histidine and of biotin. Then the mixture was added over the surface of GM agar plates and was left incubating 48 h at 37 °C. Afterwards, the colonies were counted manually [25, 28]. A compound is considered mutagenic if MI >2.0 or if compound samples contain more revertants than NQNO controls [33–36].

### *Vibrio harveyi* Antimutagenicity Assay

Procedure followed in the *V. harveyi* antimutagenicity assay [34, 35] was similar to the mutagenicity method described before. Differential point is that 10 μL of NQNO (final concentration of 40 ng/mL) were added to *V. harveyi* bacterial culture in BOSS liquid medium 15 min after the addition of tested compound to the culture. The results were expressed as the inhibition percentage of mutagenic effect, which was calculated with the formula: 100 − [(*R*_1_/*R*_2_) × 100], where *R*_1_ is the number of revertants per plate in the presence of both mutagen and tested substance, and *R*_2_ is the number of revertants per plate in the presence of mutagen [34, 36]. The antimutagenic effect was considered weak/absent, moderate, or strong when the inhibition percentage was up to 25%, from 25 to 40%, and higher than 40%, respectively [34, 37]. *S. typhimurium* antimutagenicity assay was based on similar assumptions as the *V. harveyi* antimutagenicity test. In this case, the chemopreventive activity was determined at two concentrations of a tested compounds and a standard mutagen, i.e., 40 and 500 ng/mL.

### DPPH Assay

The capacity of selected 8-methoxy-purine-2,6-dione derivatives to scavenge stable free radical DPPH was assessed [22, 38–42]. A blank and a triplicate of 10 different concentrations of each compound or of each positive control (gallic and ascorbic acids) were evaluated in the experiment. Aforesaid concentrations of compounds were obtained by dilution from a 1000 μM stock solution in methanol. One hundred microliters of DPPH stock solution were added to each well; later, 100 μL of the corresponding compound dilution were added. Consequently, the final concentration of DPPH radical was 250 μM, whereas the final concentrations of the tested compounds or positive controls were 500, 375, 250, 125, 62.5, 50, 37.5, 25, 12.5, and 6.25 μM. Afterwards, the 96-plate was wrapped with aluminum and stored in the dark 30 min prior to the spectrophotometric measurement at a 517-nm wavelength. The obtained absorbances were used to calculate the percentage of radical scavenged by the action of a compound or of a positive control at each concentration using a formula 100 × (*A*_0_ − *A*_m_)/*A*_0_. In this equation *A*_m_ is the average absorbance of the three triplicates measured at the corresponding concentration and *A*_0_ is the average absorbance of DPPH radical in the absence of the tested substance. Knowing inhibition data at the different concentrations, IC_50_ was calculated when applicable [40].

## Results

### *Cunninghamella* Biotransformation

The metabolic degradation of compounds 2–4 was studied in the *Cunninghamella* biotransformation assay, being these derivatives tested in three different *Cunninghamella* species: *C. echinulata*, *C. blakesleeana*, and *C. elegans*. Compound 1 was not evaluated within this experiment due to its structural similarity to compound 2. Metabolites were determined through the study of the corresponding LC-MS/MS spectra. Unless stated otherwise, the metabolites discussed onwards are the ones found at the end of experiment after 7-day incubation of investigated purine-2-6-dione derivatives with the corresponding *Cunninghamella* strain.

None of the three *Cunninghamella* strains evaluated generated detectable metabolites of 2 in a biotransformation assay, according to the LC-MS/MS spectra recorded. The main peak observed in LC-MS/MS corresponded with protonated molecule [M + H]^+^ of 2 (*m*/*z* 283) and was eluted in LC at a retention time of 3.52 min.

**Fig. 2:**
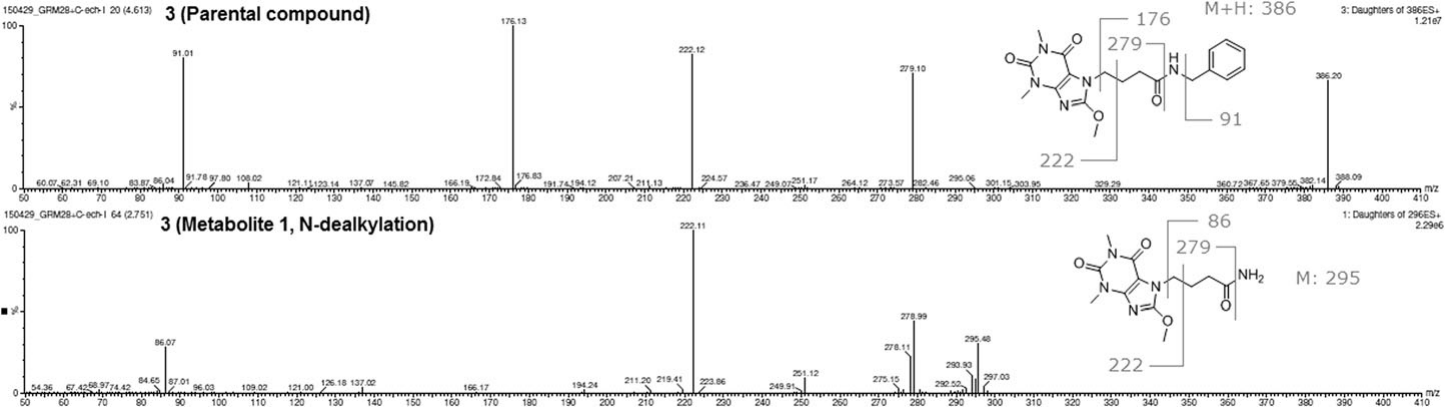
LC-MS/MS spectra compound 3 and its 3-M1 metabolite

**Fig. 3:**
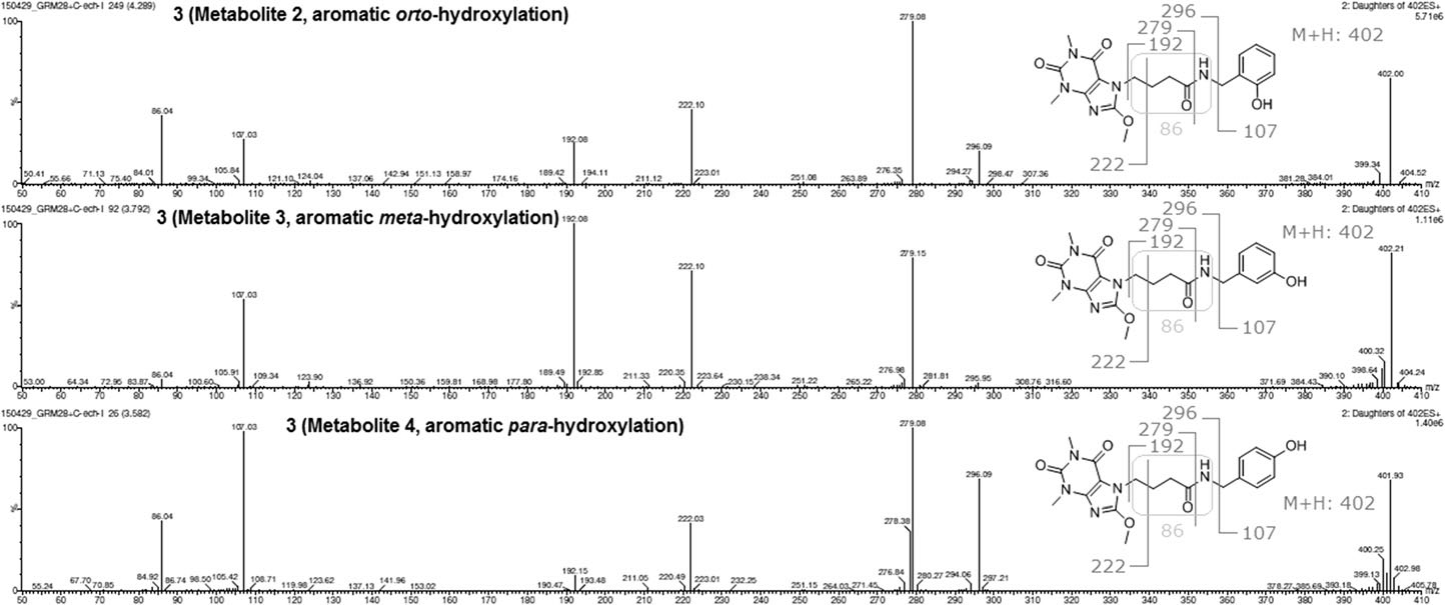
LC-MS/MS spectra of compound 3 and its metabolites 3-M2, 3-M3, and 3-M4

**Fig. 4:**
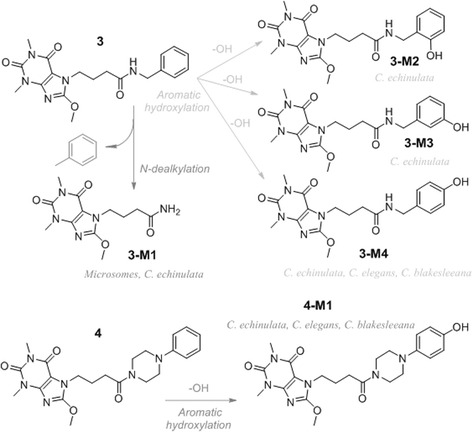
General scheme for compounds 3 and 4 biotransformation pathways in *Cunninghamella* and microsomal models

In case of 3, a protonated molecule [M + H]^+^ (*m*/*z* 386) of a parental compound (Fig. 2) remained to be the most important derivative ion present in the extract, as only a 0.6, 4.6, and 7.6% of a tested compound was metabolized at the end of experiment in *C. elegans*, *C. blakesleeana*, and *C. echinulata*, respectively (Table 1). The main metabolite (3-M4) in three strains corresponded to the product of aromatic hydroxylation (Fig. 3) of a parental compound, and was eluted at a retention time of 3.62 min (*m*/*z* 402). The percentages of the metabolite observed were 0.6, 4.6, and 4.2% in *C. elegans*, *C. blakesleeana*, and *C. echinulata*, respectively. No more metabolites were detected in the first two strains. *C. echinulata* generated three additional metabolites in the biotransformation assay. The first of these three metabolites (3-M1) had a retention time of 2.75 min, an abundance of 1.5%, and an *m*/*z* of 296. On the other hand, the main peak in MS of the two remaining metabolites had a molecular weight of 402 (Fig. 3). The percentages of appearance of these two metabolites and their retention times were 3.79 min and 0.3% for the first metabolite (3-M3) and 4.29 min and 1.6% for the second (3-M2) (Table 1, Figs. 3 and 4).

**Table 1:** Metabolites of compounds 3 and 4 detected in *Cunninghamella* biotransformation assay

Metabolite/compound				% of compound/metabolite in blank or different *Cunninghamella* strains			
Cmp.	*t*_r_ (min)	[M + H]^+^	Biotransformation reaction type	Blank	*C. elegans*	*C. blakesleeana*	*C. echinulata*
3	4.61	386	Parental compound	100.0	99.4	95.4	92.4
3-M1 3-M2 3-M3 3-M4	2.75 4.29 3.79 3.62	296 402 402 402	*N*-dealkylation Aromatic hydroxylation Aromatic hydroxylation Aromatic hydroxylation	– – – –	– – – 0.6	– – – 4.6	1.5 1.6 0.3 4.2
4	5.19	441	Parental compound	100.0	47.0	33.3	12.4
4-M1	3.37	457	Aromatic hydroxylation	–	53.0	66.6	87.6

*t*_*r*_ retention time

A protonated molecule [M + H] at *m*/*z* 441 was detected in mass spectra of four after the biotransformation processes, corresponding to a molecular weight (*m*/*z* 440) of a parental compound. In all *Cunninghamella* strains, only one metabolite (4-M1), the product of aromatic hydroxylation, was observed, at a retention time of 3.37 min and with *m*/*z* 457. The calculated percentages of the parental compound metabolized in each case were 53.0, 66.6, and 87.6% in *C. elegans*, *C. blakesleeana*, and *C. echinulata*, respectively (Table 1, Fig. 4).

### Microsomal Biotransformation

The metabolic stability of compounds 1 and 3 was measured by the incubation with rat liver microsomes and subsequent monitoring of the metabolites formed by LC-MS/MS. Compound 2 was not examined with microsomes due to its metabolic stability in a former *Cunninghamella* assay. During the microsomal biotransformation, compound 1 was metabolically stable as no metabolites were observed after 1 h of its incubation with microsomes: only a protonated paternal compound was detected in LC-MS/MS spectra.

**Fig. 5:**
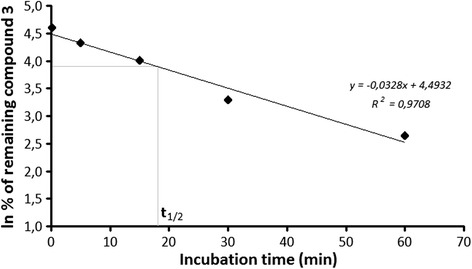
Graphical calculation of *t*_1/2_ during biotransformation in rat liver microsomes of compound 3 and its depletion along time

On the other hand, only one metabolite (3-M1) of compound 3 (Table 2) was observed after rat microsomal incubation, being its molecular weight and its retention time 296 g/mol and 2.78 min, respectively. The metabolite found was the product of the *N*-dealkylation of a parental compound (Fig. 4). Calculated in vitro half-time (*t*_1/2_) for 3 was 17.7 min, and intrinsic clearance (Cl_int_) was 97.88 μL/min/mg protein (Fig. 5).

**Table 2:** Metabolites of compound 3 observed in rat microsomal biotransformation after 30 min of incubation

Cmp.	*t*_r_ (min)	[M + H]^+^	Biotransformation reaction type	% content among metabolites
3	4.61	386	Parental compound	−
3-M1	2.75	296	*N*-dealkylation	100

*t*_*r*_ retention time

### Mutagenicity

The results of mutagenicity assay in four *Vibrio harveyi* strains are shown in Table 3. None of the evaluated four 8-methoxy-purine-2,6-dione derivatives displayed mutagenicity at the concentration of 40 ng/mL, as MI of four investigated compounds was always below 2.0 in all the *Vibrio harveyi* strains analyzed and the number of revertants counted in test samples was always lower than the values determined for NQNO controls. Moreover, all investigated compounds were also devoid of mutagenic activity according to the Ames test performed at the compound concentrations of 40 and 500 ng/mL (Table 3) using the *Salmonella typhimurium* TA100 strain.

**Table 3:** Mutagenicity of compounds 1–4 using the *Vibrio harveyi* assay and the Ames test

Number of revertants per plate												
Cmp.	*Vibrio harveyi*								*Salmonella typhimurium* TA100			
	BB7		BB7X		BB7M		BB7XM		40 ng/mL		500 ng/mL	
	Mean ± SD	MI	Mean ± SD	MI	Mean ± SD	MI	Mean ± SD	MI	Mean ± SD	MI	Mean ± SD	MI
CTR1	16 ± 4		14 ± 4		27 ± 5		21 ± 5		nd		nd	
CTR2	13 ± 3		22 ± 8		27 ± 3		33 ± 6		36 ± 6		27 ± 9	
NQNO	36 ± 7	2.8	55 ± 7	2.5	56 ± 7	2.1	75 ± 5	2.3	72 ± 10	2.0	84 ± 10	3.1
1	12 ± 2	0.9	30 ± 1	1.4	33 ± 8	1.2	37 ± 11	1.1	12 ± 5	0.3	16 ± 3	0.6
2	15 ± 2	1.2	27 ± 2	1.2	36 ± 9	1.3	53 ± 8	1.6	10 ± 3	0.3	14 ± 5	0.5
3	18 ± 6	1.4	35 ± 4	1.6	35 ± 5	1.3	35 ± 7	1.1	53 ± 11	1.5	6 ± 3	0.2
4	10 ± 4	0.8	27 ± 7	1.2	42 ± 11		41 ± 9	1.2	48 ± 7	1.3	10 ± 1	0.4

*CTR1* control sample without mutagen, *CTR2* control sample without mutagen but with DMSO, *NQNO* experiment with 4-nitroquinoline *N*-oxide, *MI* mutagenicity index, *nd* not determined

### Antimutagenicity

According to data obtained in the antimutagenicity assay (Table 4), four 8-methoxy-purine-2,6-dione derivatives inhibited the mutagenic action of a standard mutagen NQNO in four *Vibrio harveyi* strains evaluated. All derivatives exhibited a very strong chemopreventive activity in two modified *V. harveyi* strains (BB7X and BB7M). The antimutagenic percentages calculated ranged from 56 to 74%, and from 39 to 54% in BB7X and BB7M strains, respectively. In contrast, 1 (20%), 2 (38%), and 3 (29%) exerted a moderate or a close to moderate chemopreventive effect in a wild BB7 strain, whereas 4 (53%) was a strong chemopreventive agent in the same strain. Finally, chemopreventive activity exerted by the evaluated 8-methoxy-purine-2,6-dione derivatives was much lower in the BB7XM strain, in which chemopreventive percentages ranged from 9 to 15%. Moreover, all derivatives showed a very potent antimutagenic activity in *S. typhimurium* TA100 strain. The chemopreventive percentages ranged from 88 to 96% and from 88 to 95% for concentrations of 40 and of 500 ng/mL, respectively (Table 4).

**Table 4:** Antimutagenicity of compounds 1–4 against NQNO using the *Vibrio harveyi* assay and the Ames test

Number of revertants per plate												
Cmp.	*Vibrio harveyi*								*Salmonella typhimurium* TA100			
	BB7		BB7X		BB7M		BB7XM		40 ng/mL		500 ng/mL	
	Mean ± SD	%	Mean ± SD	%	Mean ± SD	%	Mean ± SD	%	Mean ± SD	%	Mean ± SD	%
CTR1	23 ± 6		28 ± 3		29 ± 7		20 ± 9		nd		nd	
CTR2	30 ± 5		27 ± 5		35 ± 7		53 ± 7		8 ± 2		11 ± 5	
NQNO	54 ± 8		108 ± 11		98 ± 11		76 ± 10		84 ± 11		95 ± 7	
1	44 ± 6	20	28 ± 7	74	60 ± 10	39	69 ± 10	10	10 ± 3	88	5 ± 3	95
2	34 ± 7	38	35 ± 6	68	45 ± 10	54	68 ± 8	12	7 ± 2	92	9 ± 4	91
3	38 ± 9	29	45 ± 9	58	48 ± 8	50	65 ± 7	15	4 ± 2	96	8 ± 3	92
4	26 ± 6	53	47 ± 10	56	50 ± 9	49	70 ± 7	9	6 ± 2	93	11 ± 3	88

Mean values from experiments ± standard deviation (SD) are presented (with inhibition of NQNO mutagenicity indicated in parentheses). Number of colonies is the average number of revertants per plate

*CTR1* control experiment without mutagen, *CTR2* control experiment without mutagen, but with DMSO, *NQNO* experiment with 4-nitroquinoline *N*-oxide as a single compound.

### Antioxidant Activity

None of the compounds 1–4 showed capacity to scavenge DPPH radical, in comparison to positive controls considered (ascorbic and gallic acids). The maximum radical scavenging percentage observed was 6.56% for 3 at the concentration of 250 μM.

## Discussion

Within the present study, in vitro biotransformation of some promising 8-methoxy-purine-2,6-dione derivatives (1–4) was investigated. Additionally, muta-, antimutagenicity, and antioxidant potency of investigated compounds were assessed.

Among the tested compounds, 1 and 2 were metabolically stable as 1 did not undergo biotransformation in rat microsomes and no metabolites of 2 were detected in the *Cunninghamella* model. *Cunninghamella* biotransformation of compounds 3 and 4 rendered as metabolites the product of aromatic hydroxylation in both compounds and *N*-dealkylation metabolite of compound 3. Nevertheless, this last metabolite was only detected in 3 with *C. echinulata*. With reference to M2 and M3 metabolites of 3, we have three possible different aromatic hydroxylation isomers: *ortho-*, *meta-* and *para-*hydroxylated metabolites. From a chemical point of view, −CH_2_-NH-R substituent present in 4 is a mild electron donating moiety which activates slightly benzene ring in electrophilic aromatic substitution. In this reaction, the added hydroxyl group is directed preferably towards *para-* and *ortho-*positions, being *meta-*position the least favored. Furthermore, the steric hindrance plays an important role, promoting *para-* direction over *ortho-*direction. Therefore, considering the percentages of metabolites and their polarity (in reverse order than retention time, and supposing that *para-* will be slightly more polar than *meta-*, and *meta-* than *ortho-*), we hypothesize that the biotransformation products at retention times of 3.62, 3.79, and 4.29 min are the products of *para-*hydroxylation (3-M4), *meta-*hydroxylation (3-M3), and *ortho-*hydroxylation (3-M2) reactions of 3, respectively. This hypothesis also explains why *para-*hydroxylated metabolite (3-M4) is the only one observed after the biotransformation of 3 in *C. elegans* and in *C. blakesleeana*. Regarding compound 4, by analogy with 3, we hypothesize that the product of its aromatic hydroxylation is *para-*hydroxylated derivative.

On the other hand, *N*-dealkylation product (3-M1) was a unique metabolite observed in microsomal biotransformation of 3, being its intrinsic clearance quite high. Consequently, 8-methoxy-purine-2,6-dione ring is the most stable part of the molecule as it does not undergo biotransformations, which are limited to 7-alkyl substituent of purine core. Probably, compounds 1 and 2 do not suffer any biotransformation because they do not contain any aromatic ring or secondary amides like derivatives 3 and 4.

Calculated in vitro half-time for 3 was 17.7 min, and intrinsic clearance was 97.88 μL/min/mg protein. This Cl_int_ is high as it is 3.09-fold lower than the 302.00 μL/min/mg established for imipramine [23]. This experimental fact means that 3 is metabolized relatively fast.

Four 8-methoxy-purine-2,6-dione derivatives evaluated in this study are safe from a mutagenic point of view, as they successfully overcome two initial screenings of their mutagenic potential (the *Vibrio harveyi* and the Ames tests) without displaying mutagenic activity. Interestingly, all the investigated compounds showed antimutagenic potential, which means that they have chemopreventive activity. Therefore, they might potentially delay, inhibit, or even reverse carcinogenesis [38]. Nevertheless, the mechanism that explains their chemopreventive action is not the free radical scavenging activity, as they showed a low or non-existent capacity to quench DPPH radical in DPPH assay performed. Thus, further research should be performed in the future to ascertain the exact mechanism responsible for this chemopreventive activity [43, 44].

To sum up, within the study, metabolic profile and selected biological properties of some novel 8-methoxy-purine-2,6-dione derivatives (compounds 1–4) with analgesic and anti-inflammatory properties were evaluated. It was demonstrated that in the *Cunninghamella* model, compound 2 did not undergo any biotransformation; whereas 3 and 4 showed less metabolic stability as they underwent biotransformation reactions. Moreover, the new derivatives of purine-2,6-dione do not possess mutagenic potential in the microbiological models considered and showed a strong chemopreventive activity in the modified *Vibrio harveyi* strains BB7X and BB7M.

Therefore, the obtained results might represent an important step in designing and planning future studies with purinediones.

## References

[1] Asha S, Vidayavathi M (2009). *Cunninghamella*—a microbial model for drug metabolism studies—a review. Biotechnology Advances.

[2] Amadio J, Murphy CD (2011). Production of human metabolites of the anti-cancer drug flutamide via biotransformation in *Cunninghamella* species. Biotechnology Letters.

[3] Pękala E, Kubowicz P, Łażewska D (2012). *Cunninghamella* as a microbiological model for metabolism of histamine H3 receptor antagonist 1-[3-(4-tert butylphenoxy)propyl]piperidine. Applied Biochemistry and Biotechnology.

[4] Watanabe S, Kuzhiumparambil U, Winiarski Z, Fu S (2016). Biotransformation of synthetic cannabinoids JWH-018, JWH-073 and AM2201 by *Cunninghamella elegans*. Forensic Science International.

[5] Pękala E, Kochan M, Carnell AJ (2009). Biotransformation of synthetic cannabinoids JWH-018, JWH-073 and AM2201 by *Cunninghamella elegans*. Letters in Applied Microbiology.

[6] Piska K, Żelaszczyk D, Jamrozik M, Kubowicz-Kwaśny P, Pękala E (2016). *Cunninghamella* biotransformation—similarities to human drug metabolism and its relevance for the drug discovery process. Current Drug Metabolism.

[7] Srisailam K, Raj Kumar V, Veeresham C (2010). Predicting drug interaction of Clopidogrel on microbial metabolism of diclofenac. Applied Biochemistry and Biotechnology.

[8] Srisailam K, Veeresham C (2010). Biotransformation of celecoxib using microbial cultures. Applied Biochemistry and Biotechnology.

[9] Amadio J, Gordon K, Murphy CD (2010). Biotransformation of flurbiprofen by *Cunninghamella* species. Applied and Environmental Microbiology.

[10] Bourdon F, Lecoeur M, Verones V, Vaccher C, Lebegue N, Dine T, Kambia N, Goossens JF (2013). In vitro pharmacokinetic profile of a benzopyridooxathiazepine derivative using rat microsomes and hepatocytes: identification of phases I and II metabolites. J. Pharmaceut. Biomed..

[11] Ahn S, Kearbey JD, Li CM, Duke CB, Miller DD, Dalton JT (2011). Biotransformation of a novel antimitotic agent, I-387, by mouse, rat, dog, monkey, and human liver microsomes and *in vivo* pharmacokinetics in mice. Drug Metabolism and Disposition.

[12] Sun H (2012). Capture hydrolysis signals in the microsomal stability assay: molecular mechanisms of the alkyl ester drug and prodrug metabolism. Bioorganic & Medicinal Chemistry Letters.

[13] Marques LM, da Silva EA, Gouvea DR, Vessecchi R, Pupo MT, Lopes NP, Kato MJ, de Oliveira AR (2014). In vitro metabolism of the alkaloid piplartine by rat liver microsomes. Journal of Pharmaceutical and Biomedical Analysis.

[14] Asha S, Vidyavathi M (2010). Role of human liver microsomes in in vitro metabolism of drugs-a review. Applied Biochemistry and Biotechnology.

[15] Zygmunt M, Chłoń-Rzepa G, Sapa J (2014). Analgesic and anti-inflammatory activity of 7-substituted purine-2,6-diones. Pharm. Rep..

[16] Zygmunt M, Chłoń-Rzepa G, Sapa J, Pawłowski M (2015). Analgesic activity of new 8-methoxy-1,3-dimethyl-2,6-dioxo-purin-7-yl derivatives with carboxylic, ester or amide moieties. Pharm. Rep..

[17] Quinn L, Dempsey R, Casey E, Kane A, Murphy CD (2015). Production of drug metabolites by immobilised *Cunninghamella* elegans: from screening to scale up. Journal of Industrial Microbiology & Biotechnology.

[18] Hamelin BA, Bouayad A, Drolet B, Gravel A, Turgeon J (1998). In vitro characterization of cytochrome P450 2D6 inhibition by classic histamine H1 receptor antagonists. Drug Metabolism and Disposition.

[19] Huang J, Si L, Fan Z, Hu L, Qiu J, Li G (2011). In vitro metabolic stability and metabolite profiling of TJ0711 hydrochloride, a newly developed vasodilatory β-blocker, using a liquid chromatography-tandem mass spectrometry method. Journal of Chromatography. B, Analytical Technologies in the Biomedical and Life Sciences.

[20] Di L, Kerns EH, Hong Y, Kleintop TA, McConnell OJ, Huryn DM (2003). Optimization of a higher throughput microsomal stability screening assay for profiling drug discovery candidates. Journal of Biomolecular Screening.

[21] Canale V, Kurczab R, Partyka A, Satała G, Słoczyńska K, Kos T, Jastrzębska-Więsek M, Siwek A, Pękala E, Bojarski AJ, Wesołowska A, Popik P, Zajdel P (2016). *N*-alkylated arylsulfonamides of (aryloxy)ethyl piperidines: 5-HT7 receptor selectivity versus multireceptor profile. Bioorganic & Medicinal Chemistry.

[22] Słoczyńska K, Pańczyk K, Waszkielewicz AM, Marona H, Pękala E (2016). In vitro mutagenic, antimutagenic and antioxidant activities evaluation and biotransformation of some bioactive 4-substituted 1-(2-methoxyphenyl)piperazine derivatives. Journal of Biochemical and Molecular Toxicology.

[23] Singh JK, Solanki A, Shirsath VS (2012). Comparative in vitro intrinsic clearance of imipramine in multiple species liver micrososmes: human, rat, mouse and dog. J. Drug Metab. Toxicol..

[24] Basavapathruni A, Olhava EJ, Daigle SR, Therkelsen CA, Jin L, Boriack-Sjodin PA, Allain CJ, Klaus CR, Raimondi A, Scott MP, Dovletoglou A, Richon VM, Pollock RM, Copeland RA, Moyer MP, Chesworth R, Pearson PG, Waters NJ (2014). Nonclinical pharmacokinetics and metabolism of EPZ-5676, a novel DOT1L histone methyltransferase inhibitor. Biopharmaceutics & Drug Disposition.

[25] Czyż A, Jasiecki J, Bogdan A, Szpilewska H, Węgrzyn G (2000). Genetically modified *Vibrio harveyi* strains as potential bioindicators of mutagenic pollution of marine environments. Applied and Environmental Microbiology.

[26] Piosik J, Ulanowska K, Gwizdek-Wiśniewska A, Czyż A, Kapuściński J, Węgrzyn G (2003). Alleviation of mutagenic effects of polycyclic aromatic agents (quinacrine mustard, ICR-191 and ICR-170) by caffeine and pentoxifylline. Mutation Research.

[27] Podgórska B, Chęć E, Ulanowska K, Węgrzyn G (2005). Optimisation of the microbiological mutagenicity assay based on genetically modified *Vibrio harveyi* strains. Journal of Applied Genetics.

[28] Podgórska B, Węgrzyn G (2006). A modified *Vibrio harveyi* mutagenicity assay based on bioluminescence induction. Letters in Applied Microbiology.

[29] Mortelmans K, Zeiger E (2000). The Ames *Salmonella*/microsome mutagenicity assay. Mutation Research.

[30] Zeiger E (2013). Bacterial mutation assays. Methods in Molecular Biology.

[31] Gulluce M, Agar G, Baris O, Karadayi M, Orhan F, Sahin F (2010). Mutagenic and antimutagenic effects of hexane extract of some *Astragalus* species grown in the eastern Anatolia region Turkey. Phytotherapy Research.

[32] Fronza G, Campomenosi P, Iannone R, Abbondandolo A (1992). The 4-nitroquinoline 1-oxide mutational spectrum in single stranded DNA is characterized by guanine to pyrimidine transversions. Nucleic Acids Research.

[33] Powroźnik B, Słoczyńska K, Canale V, Grychowska K, Zajdel P, Pękala E (2016). Preliminary mutagenicity and genotoxicity evaluation of selected arylsulfonamide derivatives of (aryloxy)alkylamines with potential psychotropic properties. Journal of Applied Genetics.

[34] Słoczyńska K, Waszkielewicz AM, Marona H (2014). Preliminary assessment of mutagenic and anti-mutagenic potential of some aminoalkanolic derivatives of xanthone by use of the *Vibrio harveyi* assay. Mutation Research.

[35] Słoczyńska K, Pękala E, Wajda A, Węgrzyn G, Marona H (2010). Evaluation of mutagenic and antimutagenic properties of some bioactive xanthone derivatives using *Vibrio harveyi* test. Letters in Applied Microbiology.

[36] Pękala E, Liana P, Kubowicz P, Powroźnik B, Obniska J, Chlebek I, Węgrzyn A, Węgrzyn G (2013). Evaluation of mutagenic and antimutagenic properties of new derivatives of pyrrolidine-2,5-dione with anti-epileptic activity, by use of the *Vibrio harveyi* mutagenicity test. Mutation Research.

[37] Kamiński K, Obniska J, Chlebek I, Liana P, Pękala E (2013). Synthesis and biological properties of new *N*-Mannich bases derived from 3-methyl-3-phenyl- and 3,3-dimethyl-succinimides. Part V. Eur. J. Med. Chem..

[38] Delarmelina JM, Dutra JC, Batittucci Mdo C (2014). Antimutagenic activity of ipriflavone against the DNA-damage induced by cyclophosphamide in mice. Food and Chemical Toxicology.

[39] Domínguez-Álvarez E, Plano D, Font M, Calvo A, Prior C, Jacob C, Palop JA, Sanmartin C (2014). Synthesis and antiproliferative activity of novel selenoester derivatives. European Journal of Medicinal Chemistry.

[40] Martins M, Arantes S, Candeias F, Tinoco MT, Cruz-Morais J (2014). Antioxidant, antimicrobial and toxicological properties of *Schinus molle* L. essential oils. Journal of Ethnopharmacology.

[41] Arora DS, Chandra P (2011). In vitro antioxidant potential of some soil fungi: screening of functional compounds and their purification from *Penicillium citrinum*. Applied Biochemistry and Biotechnology.

[42] Tarhan L, Nakipoğlu M, Kavakcioğlu B, Tongul B, Nalbantsoy A (2016). The induction of growth inhibition and apoptosis in HeLa and MCF-7 cells by *Teucrinumsandrasicum*, having effective antioxidant properties. Applied Biochemistry and Biotechnology.

[43] Watanabe M, Kobayashi H, Ohta T (1994). Rapid inactivation of 3-chloro-4-(dichloromethyl)-5-hydroxy-2(5H)-furanone (MX), a potent mutagen in chlorinated drinking water, by sulfhydryl compounds. Mutation Research.

[44] Słoczyńska K, Powroźnik B, Pękala E, Waszkielewicz AM (2014). Antimutagenic compounds and their possible mechanisms of action. Journal of Applied Genetics.

